# Surveillance for Anthrax Cases Associated with Contaminated Letters, New Jersey, Delaware, and Pennsylvania, 2001

**DOI:** 10.3201/eid0810.020322

**Published:** 2002-10

**Authors:** Christina G. Tan, Hardeep S. Sandhu, Dana C. Crawford, Stephen C. Redd, Michael J. Beach, James Buehler, Eddy A. Bresnitz, Robert W. Pinner, Beth P. Bell, the Regional Anthrax Surveillance Team, the Centers for Disease Control and Prevention New Jersey Anthrax Surveillance Team

**Affiliations:** * Centers for Disease Control and Prevention, Atlanta, Georgia, USA; † New Jersey Department of Health and Senior Services, Trenton, New Jersey, USA

**Keywords:** *Bacillus anthracis*, anthrax, surveillance, bioterrorism

## Abstract

In October 2001, two inhalational anthrax and four cutaneous anthrax cases, resulting from the processing of *Bacillus anthracis*–containing envelopes at a New Jersey mail facility, were identified. Subsequently, we initiated stimulated passive hospital-based and enhanced passive surveillance for anthrax-compatible syndromes. From October 24 to December 17, 2001, hospitals reported 240,160 visits and 7,109 intensive-care unit admissions in the surveillance area (population 6.7 million persons). Following a change to reporting criteria on November 8, the average of possible inhalational anthrax reports decreased 83% from 18 to 3 per day; the proportion of reports requiring follow-up increased from 37% (105/286) to 41% (47/116). Clinical follow-up was conducted on 214 of 464 possible inhalational anthrax patients and 98 possible cutaneous anthrax patients; 49 had additional laboratory testing. No additional cases were identified. To verify the limited scope of the outbreak, surveillance was essential, though labor-intensive. The flexibility of the system allowed interim evaluation, thus improving surveillance efficiency.

In the fall of 2001, a multistate investigation involving local, state, and federal public health and law enforcement authorities identified letters intentionally contaminated with *Bacillus anthracis* spores; these letters were processed through the Trenton Processing and Distribution Center on September 18 and October 9. On October 13, the New Jersey Department of Health and Senior Services (NJDHSS) received reports of two postal employees with clinical symptoms compatible with cutaneous anthrax; their illnesses began on September 26 and 28. On October 18, following the confirmation of the first anthrax case in New Jersey, the Trenton Processing and Distribution Center was closed. Subsequently, NJDHSS identified a total of six anthrax cases (two inhalational, four cutaneous), all reported from October 13 to 24 (1,2).

On October 24, NJDHSS and the Centers for Disease Control and Prevention (CDC) began formal surveillance for specified clinical syndromes compatible with anthrax. Surveillance was implemented with the objectives of improving case finding, describing the spectrum of clinical signs and symptoms of possible anthrax illness, characterizing the population at risk, and determining the magnitude of the outbreak. This report describes the surveillance efforts and results.

## Methods

From October 24 to December 17, NJDHSS and CDC implemented passive surveillance (3) for syndromes compatible with anthrax, supported with specific laboratory testing for *B. anthracis*. This surveillance included two components: stimulated passive hospital-based surveillance (4) for inhalational anthrax and enhanced passive surveillance for inhalational anthrax and cutaneous anthrax.

### Stimulated Passive Hospital-Based Surveillance for Inhalational Anthrax

**Figure 1:**
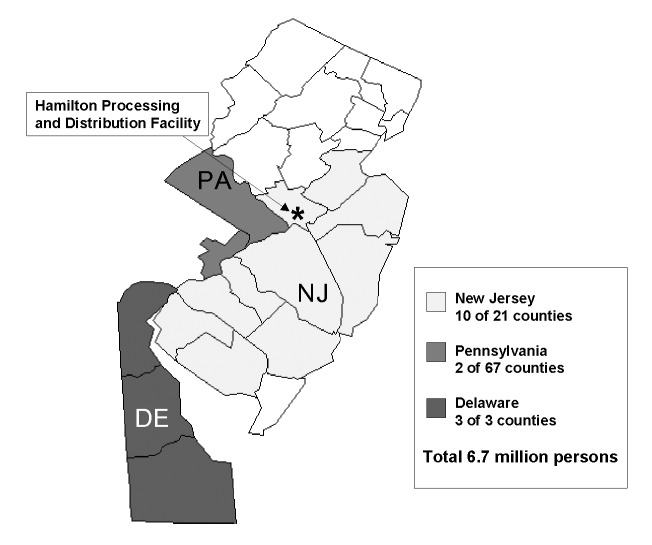
Counties participating in active surveillance, New Jersey, Pennsylvania, and Delaware, 2001.

We implemented stimulated passive hospital-based surveillance in 15 counties in New Jersey, Delaware, and Pennsylvania, on October 24. Infection control professionals (ICPs) of all acute-care hospitals of 10 New Jersey, 2 Pennsylvania, and 3 Delaware counties were invited to participate; specialty and psychiatric hospitals were not included in surveillance (Figure 1). Reporting criteria for possible inhalational anthrax included any emergency department patient with a diagnosis of respiratory failure or severe respiratory distress or any intensive-care unit (ICU) patient from whom blood, cerebrospinal fluid, or pleural fluid cultures were obtained. Reporting criteria and forms were distributed to ICPs and local and state health departments by e-mail or fax. We requested that ICPs provide a daily summary report that documented the total number of emergency department visits and ICU admissions and the total number of emergency department visits and ICU admissions that met reporting criteria for possible inhalational anthrax. For each patient whose illness met reporting criteria for inhalational anthrax, ICPs completed a case ascertainment form that provided details on the patient's demographic information and clinical symptoms.

We requested that hospitals provide daily summary reports within 1 day of the date of the reported data. Infection control professionals faxed or e-mailed completed summary and case ascertainment reports to officials in appropriate local and state health departments; these reports were then forwarded to the CDC New Jersey Emergency Operations Center at NJDHSS for review. Data summaries were provided periodically to ICPs during the surveillance period.

To improve the surveillance system, we performed interim evaluations and conducted periodic conference calls with local and state health departments and participating ICPs to gain feedback on surveillance methodology. In response to comments that initial inhalational anthrax reporting criteria encompassed a broad spectrum of differential diagnoses including illnesses unlikely to be undiagnosed anthrax, such as chronic pulmonary disease, we modified clinical criteria for reporting on November 8. ICPs were then requested to report any emergency department or ICU patient with illness onset after September 18 with 1) fever, cough, abnormal chest x-ray, and no prior chronic pulmonary disease, 2) fever, respiratory failure, or severe respiratory distress not clearly attributable to a previously diagnosed chronic pulmonary or cardiac disease, or 3) sepsis of unknown origin. We distributed revised reporting criteria to ICPs and local and state health departments through e-mail, facsimile, and telephone communication.

### Passive Surveillance for Inhalational or Cutaneous Anthrax

Passive surveillance for possible inhalational anthrax and cutaneous anthrax cases was conducted statewide in New Jersey. Reporting criteria for possible cutaneous anthrax included persons with a suspicious lesion including an ulcer with surrounding erythema, edema, or vesicles, or a blackened eschar forming 3–7 days after the onset of the skin lesion; an ulcerative or necrotic lesion and a history of possible exposure to anthrax, including employment at a postal facility or handling mail in another setting; or laboratory evidence suggestive of *B. anthracis* infection.

Reporting criteria for both inhalational anthrax and cutaneous anthrax were made available on websites of the Medical Society of New Jersey (available at: http://www.msnj.org/), New Jersey Association of Family Physicians (available at: http://www.njafp.org/), and NJDHSS (available at: http://www.state.nj.us/health/); surveillance information was also distributed through press releases to the media.

Suspicious illnesses were reported to the New Jersey Emergency Operations Center and to the CDC New Jersey Operations Center. Nurses, physicians, and scientists from NJDHSS and CDC fielded general and medical inquiries and reviewed reports.

### Clinical Follow-Up

After reviewing all surveillance reports, we followed up on reports of patients considered to be at risk based on clinical symptoms or exposure history (e.g., employment at a postal facility and occupations that involved mail handling) or of patients without clear alternative diagnoses. Through interviews with physicians, nurses, ICPs, hospital laboratory staff, and patients, we obtained additional history on clinical symptoms, exposure, and occupational history and any preliminary hospital laboratory results available by the time of follow-up.

Clinical specimens, including whole blood, sera, pleural fluid, and skin biopsies, were obtained from persons with highly suspicious illness or credible exposure history and cultured at the New Jersey Public Health and Environmental Laboratory. CDC laboratories performed additional tests, including immunohistochemical staining of clinical specimens with *B. anthracis* capsule and cell-wall antibody, *B. anthracis*-specific polymerase chain reaction, and serologic detection of immunoglobulin G to *B. anthracis* protective antigen.

### Resources Required for Surveillance

To examine the resources for surveillance, we documented the number and type of persons and organizations and the time required to collect and analyze surveillance data. We also designed and distributed a survey to describe the resources available to hospitals participating in stimulated passive surveillance and to assess ICPs’ experiences with surveillance activities. ICPs faxed completed questionnaires to the NJDHSS for analysis.

### Medical Examiner Data Review

Concurrent with the surveillance efforts, the New Jersey State Medical Examiner asked all county medical examiners to retrospectively review all unexplained deaths due to acute respiratory illness back to September 1. In addition, all county medical examiners were instructed to accept for autopsy any unexplained deaths due to acute respiratory illness.

### Data Management and Analysis

For each participating hospital, we calculated the ratio between the number of daily reports received and the number of expected reports. The expected number of reports per hospital was the number of days a hospital participated in surveillance, calculated from the date of the first report received to December 17.

We used Access 2000 (Microsoft Corp., Redmond, WA) to maintain data and generate summary reports. Data were analyzed using Epi Info 2000, Epi Map version 2, and SAS version 8.0 (SAS Institute, Inc., Cary, NC).

## Results

### Stimulated Passive Hospital-Based Surveillance

**Figure 2:**
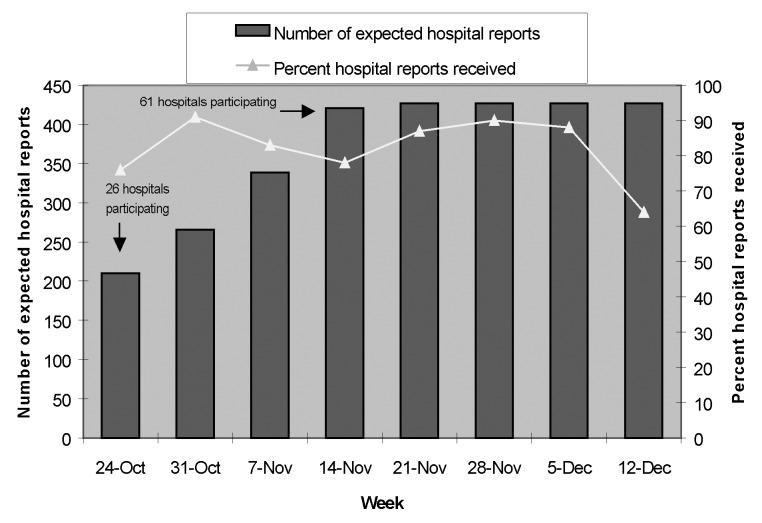
Hospital participation in passive sentinel surveillance for possible inhalational anthrax by surveillance week; Delaware, New Jersey, and Pennsylvania; October 24–December 17, 2001.

In the three states affected, all 61 acute-care hospitals from 15 counties did stimulated passive surveillance. During the 1st week of surveillance, all 26 hospitals in six counties were incorporated into the surveillance system. By the 4th week, as surveillance expanded into additional counties, all hospitals in these areas were incorporated into the system. Seventy-eight percent to 91% of all participating hospitals provided daily summary reports during 6 of 8 weeks of the surveillance period. Reporting rates were lowest during the 1st and last weeks of the surveillance interval (Figure 2).

During the entire 8-week surveillance period, in New Jersey, participating hospitals provided reports a mean of 89% (range 18% to 100%) of days during which they participated in surveillance; in Delaware, the mean was 86% (range 82% to 91%); and in Pennsylvania, the mean was 74% (range 23% to 94%).

Thirty-nine (64%) of participating hospitals were community-based acute-care facilities; among community-based facilities, participating hospitals provided reports a mean of 88% (range 18% to 100%) of the days during which they participated in surveillance. Twenty-two (36%) participating hospitals were university-based acute-care facilities; participating hospitals provided reports a mean of 84% (range 23% to 100%) of days during which they participated in surveillance.

Following an increase in reporting criteria specificity on November 8, the average of possible inhalational anthrax reports decreased 83% from 18 to 3 per day. The proportion of reports requiring follow-up increased 10% from 37% (105/286) to 41% (47/116).

### Reporting of Possible Inhalational Anthrax

During October 24 to December 17, stimulated passive hospital-based surveillance generated reports of 240,160 emergency department visits and 7,109 ICU admissions from a surveillance population of 6.7 million residents. Of these emergency department visits and ICU admissions, 402 patients whose illnesses met clinical criteria for possible inhalational anthrax were identified by ICPs. The clinical investigation team then identified 152 patients whose clinical presentation warranted collection of additional information, of whom 10 (7%) had additional laboratory testing performed at the state or CDC laboratories. Passive surveillance generated a total of 62 reports of patients meeting clinical criteria for inhalational anthrax from over 6,000 phone calls to the New Jersey Emergency Operations Center and the CDC New Jersey Operations Center. After preliminary follow-up of all 62 reports, the CDC or state laboratories performed additional tests on specimens from 13 (21%) of these patients.

No additional inhalational anthrax cases were identified among the 214 reports of possible inhalational anthrax that were followed up. A total of 103 (48%) had follow-up diagnoses of chronic pulmonary or acute infectious processes, including asthma and chronic obstructive pulmonary disease exacerbations (13 reports), bronchitis (6 reports), pneumonia (40 reports), and other pulmonary conditions (44 reports). For the remaining 111, the diagnosis of anthrax was ruled out but no alternative diagnosis was identified.

### Reporting of Possible Cutaneous Anthrax Illness

No new cutaneous anthrax cases were identified among the 98 reports meeting surveillance criteria for cutaneous anthrax, including 26 (27%) that warranted additional testing. Of these 98 reports, 32 (33%) involved follow-up diagnoses of cellulitis (6 reports), herpes zoster (5 reports), contact dermatitis (11 reports), or other dermatologic illnesses, including chronic conditions such as eczema (10 reports). For the remaining 66, the diagnosis of anthrax was ruled out, but no alternative diagnosis was identified.

### Resources Required for Surveillance

In the private sector of the population in these states, hundreds of clinicians, including ICPs and physicians, reported suspicious illnesses during the surveillance period. In the public sector, one to three epidemiologists from the NJDHSS and CDC team reviewed reports daily and entered them into a database on the day they were received. These epidemiologists then determined which reports were forwarded to the clinical investigation team for additional follow-up.

Two to three other physician epidemiologists followed up with physicians and ICPs to determine which of these patients warranted more definitive testing at the state or CDC laboratories. These physicians provided consultative information on clinical questions related to anthrax and instructed community clinicians on laboratory testing protocols.

Finally, one epidemiologist managed laboratory matters, including arrangements for transporting, tracking, and updating results for specimens. Numerous state and CDC lab staff performed testing; several epidemiologists in Atlanta helped report and interpret CDC testing results.

A total of 37 (61%) ICPs from the 61 hospitals participating in stimulated surveillance completed a survey to describe the resources available to hospitals and to assess experiences with surveillance activities. Most respondents represented community-based hospitals, with <100 beds and at least one full-time ICP. All hospitals responding in the survey had both e-mail and fax capacity; 35 (95%) ICPs received surveillance information by either fax or e-mail; the remainder received information through telephone or other contact.

Before modifications to reporting, 21 (57%) ICPs reported that each daily summary report took 0.5–1 h to complete; 10 (26%) spent 1–3 h; and 5 (13%) spent >3 h. Nine (24%) respondents stated the initial criteria were broad and included many persons with illnesses not attributable to anthrax (e.g., asthma, congestive heart failure). After modifications, 30 (81%) ICPs spent 0.5–1 h completing daily summary reports; 7 (18%) spent 1–3 h.

### Medical Examiner Data

During the surveillance period, only one unexplained death after September 1 was reported to the state medical examiner. The patient, a 44-year-old woman with a smoking history and several days of nonfebrile respiratory illness, died on October 14; chest radiographs and blood and sputum cultures were negative. She had been unemployed and had no history of mail handling. No samples were available for additional testing, and no additional follow-up was needed.

## Discussion

Intensive and comprehensive surveillance was an essential component of the national response to the crisis precipitated by this event. In New Jersey, the source of all recognized letters containing *B. anthracis*, we implemented surveillance for clinical syndromes compatible with inhalational or cutaneous anthrax over a wide geographic area representing a large population base. The information gathered through this surveillance was pivotal in documenting the relatively limited scope of the outbreak-associated anthrax cases, which in turn confirmed that exposures sufficient to cause disease were limited to persons with occupational exposure to mail processed by one distribution center.

Surveillance efforts were successful in engaging hospitals and health-care providers to identify and report patients with clinical syndromes compatible with inhalational or cutaneous anthrax. We were able to investigate the etiologies of these patients’ illnesses and document that additional cases of anthrax did not occur. This finding, in the context of a comprehensive surveillance system, helped to characterize the outbreak, demonstrating that it was confined to the originally recognized cases and confirming that the risk of developing illness in the general population was low. The finding also provided a level of assurance that cases due to this bioterrorist attack, as well as possible additional attacks on other mail processing centers in the area, were not occurring and confirmed that additional public health control measures were not needed.

This surveillance program included several successful elements. The mobilization of state and local health departments in regional efforts allowed for the monitoring of a large geographic area and fostered cooperation among the jurisdictions. We involved hospital-based surveillance participants by providing feedback and soliciting their input, and the system became more efficient with modifications implemented in response. Surveillance heightened awareness among the practicing physicians, and their cooperation allowed for timely reporting and efficient clinical follow-up.

Because all acute-care hospitals in the selected areas participated fully, providing us with reports of many patients with the defined clinical syndromes, cases in the region were likely not missed. In addition, reporting and clinical follow-up were conducted in a timely fashion, which is critical to public health surveillance and response (5,6). Daily summary reports were usually received within 1 to 2 days of the date of the reported data; longer lag times or missed reports occurred mainly during weekends. Possible inhalational anthrax reports were usually received within 1 day of the reported date of hospital visit or admission. Follow-up of each possible inhalational anthrax report took up to several days to complete; lag time in most circumstances was attributable to the period of time needed to receive laboratory results.

Our surveillance efforts had several limitations. While hospital-based surveillance was limited to selected counties, numerous reports throughout New Jersey were received. Cases before surveillance implementation may have been missed, but the state medical examiner’s retrospective data would have likely captured these possible earlier cases. Finally, surveillance was costly because of demands on personnel in participating hospitals and at the health department, which was dependent on support from CDC personnel assigned to the state during the outbreak period. This intense level of surveillance was justifiable given the nature of the anthrax emergency, but it was neither necessary nor feasible to sustain in the long term, once information showed that no additional anthrax exposures had occurred and the upper limit of the incubation period had passed. Keeping the resource-intensiveness in mind, the best ways to integrate surveillance of bioterrorist attacks into existing public health systems need to be evaluated.

Because the agents or methods of future bioterrorist attacks cannot be predicted with certainty, planning surveillance to detect a future bioterrorist attack will require that public health departments consider all possible scenarios and develop a multifaceted approach (7). A future attack might be similar to the recent experience with *B. anthracis*, in which astute clinicians reported a small number of cases, illustrating how community health-care providers are integral to successful surveillance efforts. In this scenario, detecting new cases will depend on accurate diagnostics and timely reporting by medical care providers, highlighting the importance of educating practicing clinicians on what to report, how to report, and ultimately how to interface with the public health system. To this end, public health departments should foster education about bioterrorism and surveillance in the medical community and engage key community medical personnel in these educational and surveillance efforts (8). In addition, public health departments should encourage clinicians to report diagnostic clues and patients with illness patterns that might indicate an unusual infectious disease outbreak associated with intentional release of a biologic agent (9). Finally, public health departments should develop the capability to immediately investigate suspicious reports (10). As our experience in New Jersey demonstrates, establishing and maintaining a comprehensive surveillance system in response to a bioterrorist attack is complex and resource-intensive. Once our surveillance system established that the outbreak was not ongoing, sustaining such an intense surveillance effort was not necessary. The greater challenge for public health departments in the United States will be to design sustainable systems that can assist in detecting future outbreaks.
